# Autologous microfragmented adipose tissue and leukocyte-poor platelet-rich plasma combined with hyaluronic acid show comparable clinical outcomes for symptomatic early knee osteoarthritis over a two-year follow-up period: a prospective randomized clinical trial

**DOI:** 10.1007/s00590-022-03356-2

**Published:** 2022-08-23

**Authors:** Alberto Gobbi, Ignacio Dallo, Riccardo D’Ambrosi

**Affiliations:** 1 Orthopaedic Arthroscopic Surgery International (OASI) Bioresearch Foundation Gobbi NPO, Milan, Italy; 2 IRCCS Istituto Ortopedico Galeazzi, Milan, Italy; 3 Dipartimento Di Scienze Biomediche Per La Salute, Università Degli Studi Di Milano, Milan, Italy

**Keywords:** Leucocyte-poor platelet-rich plasma, Microfragmented adipose tissue, Knee, Osteoarthritis, LP-PRP, Hyaluronic acid

## Abstract

**Purpose:**

The purpose of this prospective randomized clinical trial is to compare the clinical outcomes of three injections of leucocyte-poor platelet-rich plasma (LP-PRP) and hyaluronic acid (HA) to a single dose of autologous microfragmented adipose tissue (AMAT) in patients with mild osteoarthritis at a two-year follow-up.

**Methods:**

Eighty symptomatic knees in fifty patients (mean age: 62.38 ± 11.88 years) with Kellgren-Lawrence grade 0 to 2 osteoarthritis were non blinded, randomly allocated into two equal groups. Group 1 consisted of 40 knees that received autologous LP-PRP + HA; Group 2 consisted of 40 knees treated with a single dose of AMAT injection. The outcomes were measured by Tegner, Marx, Visual Analogue Scale (VAS) for pain, International Knee Documentation Committee, and Knee Injury and Osteoarthritis Outcome Score (KOOS) at 6 (T_1_), 12 (T_2_), and 24 (T_3_) months. Adverse events were recorded at each follow-up timepoint. To assess score differences among subjects of the same gender and age, a subgroup analysis was performed.

**Results:**

Both groups had significant clinical and functional improvement at 6, 12, and 24 months (*p* < 0.05). Comparing the two groups, the AMAT groups showed significantly higher pre-operative Marx score (3.35 ± 4.91 vs. 1.78 ± 3.91) and VAS score (5.03 ± 2.02 vs. 3.85 ± 1.68) (*p* < 0.05), higher VAS (3.89 ± 2.51 vs. 2.64 ± 2.00) at *T*_2_ and KOOS-ADL (79.60 ± 20.20 vs. 65.68 ± 23.62), and lower KOOS-Sports (50.30 ± 30.15 vs. 68.35 ± 30.39) at T_3_ (*p* < 0.05). No patient from either group had experienced major adverse effects. In the LP-PRP group 12 (30%) patients presented swelling, redness, and mild pain for one day after injection and two patients had synovitis for two days and required paracetamol and local ice. In AMAT group 5 (12.5%) patients had ecchymosis and bruising at the fat aspiration site for three days.

**Conclusion:**

AMAT did not show significant superior clinical improvement compared with three LP-PRP combined with HA injections in terms of functional improvement at different follow-up points. Both procedures were safe with no major complications reporting good results at mid-term follow-up, improving knee function, pain, and quality of live regardless of age and gender.

**Level of evidence:**

Level I—Prospective Randomized Clinical Trial.

**Supplementary Information:**

The online version contains supplementary material available at 10.1007/s00590-022-03356-2.

## Introduction

There is a growing recognition of the importance of identifying the early stages of degenerative processes in knee osteoarthritis (OA), the period of the disease during which there may still be some ability to regenerate articular cartilage, which is permanently lost in the late stage of the disease [1, 2]. A definition of the early stage of OA is important for the proper identification and treatment of patients at risk of progression, enabling better design of trials for assessing the potential and indications of available and new treatments, and therefore better allocate resources and manage patients affected by symptomatic lesions of knee cartilage [1, 2].

Hyaluronic acid (HA) is a polysaccharide, formed from numerous disaccharide subunits of D-glucuronic acid and N-acetylglucosamine, which belongs to the glycosaminoglycan family [3]. In joints, hyaluronic acid plays a key role in maintaining close functional and metabolic interdependencies between synovial membrane, synovial fluid, articular cartilage, and indirectly subchondral bone [3]. In vitro studies have shown that hyaluronic acid is able to interact with several cellular receptors, modulating both acute and chronic inflammatory processes. Intra-articularly administered HA aims to positively affect both the symptomatology and delayed progression of OA through anti-inflammatory, chondroprotective, analgesic, and stimulating effects on the production of endogenous HA [3].

Platelet-rich plasma (PRP) represents a simple, inexpensive and minimally invasive option for obtaining a concentrate of autologous-derived growth factors and other bio-active molecules capable of stimulating tissue healing and regeneration, as well as anti-inflammatory and anti-catabolic molecules [4]. Pre-clinical studies have shown promising results, supporting the use of PRP also for the treatment of OA [4]. Indeed, by infiltrative application, PRP acts on the entire joint environment with a homeopathic action, as can be seen from the effects exerted in vitro on different cell types (synovial, stem, and meniscus) and in vivo in different pathological models (focal or degenerative lesions) [4].

When PRP and HA are used in combination these effects are enhanced and prolonged. HA creates a bioactive scaffolding in which the platelets progressively release their growth factors [5]. Regen PRP does not negatively affect the mechanical, elastic or viscous properties of HA [5]. Adipose-derived stem cells (ADSCs) have attracted attention in recent years due to their high availability and less invalidity of the harvesting procedure [6]. In addition to their ability to differentiate, i.e., acquire the phenotype of the cells of the tissue to be treated, it has been shown in recent years that the therapeutic potential of adipose-derived stem cells lies primarily in their ability to interact with the surrounding microenvironment [7]. Numerous in vitro and proclitic studies have, in fact, shown how these mechanisms are able to modulate the activity of resident cells, leading to an improvement, or in some cases a restoration of tissue/organ homeostasis [6, 7]. Some clinical in vivo human studies were performed, which have shown encouraging results in the treatment of OA [6, 7]

Given that all these modalities are potentially promising, this study primarily aimed to compare clinical outcomes of a single dose of autologous microfragmented adipose tissue (AMAT) against three repeated doses of leucocyte-poor platelet-rich plasma (LP-PRP) in association with HA in the treatment of mild symptomatic knee OA; the secondary purpose is to assess whether demographic factors such as age and sex may affect the final outcome; finally to evaluate the adverse reactions of the two treatments. Currently, only one study reported one-year follow-up comparing AMAT versus LP-PRP + HA [8]. It was hypothesized that a single AMAT injections will improve patients’ quality of life and functional status and will decrease pain level significantly more than LP-PRP + HA injections in patients with symptomatic early knee OA with a 24-month follow-up.

## Materials and methods

### Study design

In this prospective randomized trial, patients were recruited from November 2015 to December 2017. This study has been approved by the Institutional Review Board OASI Foundation and conforms to Helsinki and Good Clinical’s declaration Practice: Consolidate Guideline. The study was conducted following the CONSORT Guidelines for randomized clinical trials (RCT) and has been registered in the Research Registry [9].

### Eligibility criteria

All patients were provided with a specific written consent form to sign before treatment. Detailed inclusion and exclusion criteria are reported in Table 1. Pre-operative radiographs were evaluated according to the Kellgren-Lawrence OA classification [10]. Each patient was studied using a standing anteroposterior (AP) long-leg radiograph, standing AP and lateral views, skyline patellofemoral, and standing 45° flexion knee views. Furthermore, magnetic resonance imaging (MRI) was also performed in all treated knees [11]. Mild knee OA was classified based on radiographic findings as Kellgren-Lawrence grade 0 to 2. A complete hematology screening was performed before testing.

**Table 1:** Inclusion and exclusion criteria

*Inclusion criteria*
1. Symptomatic knee osteoarthritic (Kellgren-Lawrence Grade 1–2 cartilage lesions on radiographs or early OA on MRI)
2. Aged over 40 years with BMI < 30 kg/m^2^
3. Pain without relief with oral anti-inflammatory agents > 3 months
4. Patients with stable knees without malalignment
5. Patients who consented to either treatment modality as per the protocol
6. Normal blood results and coagulation profile (Platelets between 150,000 and 450,000/uL)
7. Patients who had not undergone any surgery on the affected knee in the 2 years before enrolment into the study
*Exclusion criteria*
1. Tricompartmental osteoarthritis, rheumatoid arthritis, or concomitant severe hip osteoarthritis
2. Previous High Tibial Osteotomy or cartilage transplantation
3. Patients with blood diseases, systemic metabolic disorders, immunodeficiency, Hepatitis B or C, HIV positive status, local or systemic infection
4. Ingestion of anti-platelet medications within 7 days before the treatment, or intra-articular or oral corticosteroids in the 3 months before initiating therapy
5. Smokers
6. Inflammatory arthritis
7. Severe cardiovascular disease

### Allocation and procedures

Randomization was performed using an online software (www.randomization.com) and patients were randomly assigned at a 1:1 ratio to 1 of 2 treatment groups [12].

#### Leucocyte-Poor Platelet-Rich Plasma (LP-PRP) in association with Hyaluronic Acid (HA)

The first group received a LP-PRP + HA injection into the osteoarthritic knee (Cellular Matrix; Regen Lab, Switzerland). One cycle involved three injections one month apart. For PRP preparation, 6 mL of whole blood were obtained by venipuncture from the antecubital vein and centrifuged for 5 min at 1,500* g* relative centrifugal field and 3,500 revolutions per minute. A mix was prepared of PRP with HA at a concentration of 3 mL of PRP for every 2 mL of HA. As per the PAW classification system, the final PRP product was classified as P2 Bβ [13–15]. The system provides a 1.6–1.8-fold increase in platelets [16–18]. PRP was active just before injection.

### Autologous microfragmented adipose tissue preparation (AMAT)

The second group received a single dose of AMAT (Lipogems International S.p.A., Milan, Italy). After aseptic precautions and under local anesthesia, adipose tissue was harvested with an abdominal lipo-harvest procedure. The subcutaneous fat was injected with up to 300 ml of tumescent fluid. Then,, up to 60 ml of adipose tissue and tumescent fluid were retrieved through a 4 mm lipoaspirate cannula and collected within a sterile medical grade single-use Shippert Tissu-Trans Collection filter (Shippert Medical Technologies, Colorado, USA). The lipoaspirate was transferred directly to an AMAT device. This is a full-immersion, and low-pressure cylindrical system in order to obtain fluid with a concentrated population of pericytes signaling cells [19]. The processed fat is subjected only to slight mechanical forces, with no detrimental effects.

### Intra-articular injection

For both groups A single physician, with more than 5 years of experience in intra-articular knee injections, performed all procedures in this study using a supra-patellar approach. The injecting physician was not involved in clinical assessment of the patients. After treatment, weight bearing was allowed in both groups; ice application was recommended for the next 24 h (15 min every 3 h) [20, 21].

### Rehabilitation protocol

Vigorous knee exercises were discouraged for the next 2 days. During all the follow-up period, was recommended to patients to perform isometric knee exercises [22]. In case of pain, redness or swelling, it has been suggested that patients use acetaminophen by mouth with a maximum dosage of up to 3000 mg per day (1000 mg every 8 h).

### Outcomes of interest

All patients were evaluated by two blinded-independent clinicians not involved in the procedure.

Subjects’ demographics (age and sex) were recorded at the screening visit after study enrollment was confirmed. Clinical outcomes were assessed by means of the Knee Injury and Osteoarthritis Outcome Score (KOOS), Visual Analogue Scale (VAS), Marx Knee Measure, and Tegner scoring systems [23–26]. The patients completed questionnaires and all scores were tabulated before the commencement of treatment (T_0_), at 6 (T_1_), 12 (T_2_), and 24 (T_3_) months after treatment.

### Complications and adverse events

Undesirable clinical developments which were not present at baseline or which increased in severity after treatment were classified as Adverse Events (AEs). Duration, type, and severity of adverse events (AEs) are recorded as defined in Table 2 [27].

**Table 2:** Definitions of adverse event (AE) severity with required interventions

Severity	Definition	Additional medication required?	Physicians advice required?
Mild	Minor discomfort noticed but does not interfere with normal daily activity	No	No
Moderate	Discomfort reducing or affecting normal daily activity	Yes	Potentially
Severe	Incapacitating with inability to work or perform normal daily activity	Yes	Yes
Serious	Permanent damage, life-threatening or death	Yes	Yes

Injections-related complications were defined as any deviation from the normal postoperative course due to the implants [28].

### Sample size estimation

The sample analysis was conducted on the primary outcome of the study (i.e., clinical outcomes as IKDC/KOOS), given the presence in literature of several studies on the effects of regenerative treatment injections on knee function. Starting from two homogeneous groups with similar IKDC/KOOS values at the baseline, a sample of 78 knees—39 for each group—was estimated to be adequate to detect a 10-point difference of IKDC/KOOS score between LP-PRP + HA and AMAT groups, assuming a standard deviation (SD) of 15, an 80% power and a 5% type I error, and using the Wilcoxon-Mann Whitney test. The estimated sample also has an 80% power to detect a five-point-score difference between time points, assuming an SD of 15, a 5% alpha, and using the Wilcoxon signed rank-sum test. To ensure statistical significance in the case of unexpected events, we recruited 40 patients for each group.

### Statistical analysis

The summary statistics were presented by absolute numbers and percentages or means and SDs. A comparison between the LP-PRP + HA and AMAT was performed with a chi-square test for categorical variables and/or t-test or Wilcoxon-Mann Whitney test, based on continuous variable distribution. Additionally, mean scores were compared at each point of time: preoperative (T_0_), at T_1_, at T_2_, and at T_3_ between the two groups. Furthermore, to compare the same score between the time points within the same group, we performed a repeated measure mixed model with a first-order autoregressive covariance structure of errors. In case of a deviation from normality assumption, a Wilcoxon signed rank-sum test was performed. Bonferroni correction was applied for multiple comparisons [29]. To assess score differences among subjects of the same gender and age, a subgroup analysis was performed. Age groups were defined with the dichotomizing age at its average higher rounded value. Correlations among scores and socio-demographic variables were estimated and tested. A two-tailed *p*-value less than 0.05 was considered statistically significant. Statistical analyses were performed using the SAS System Version 9.

## Results

### Patient recruitment

**Fig. 1:**
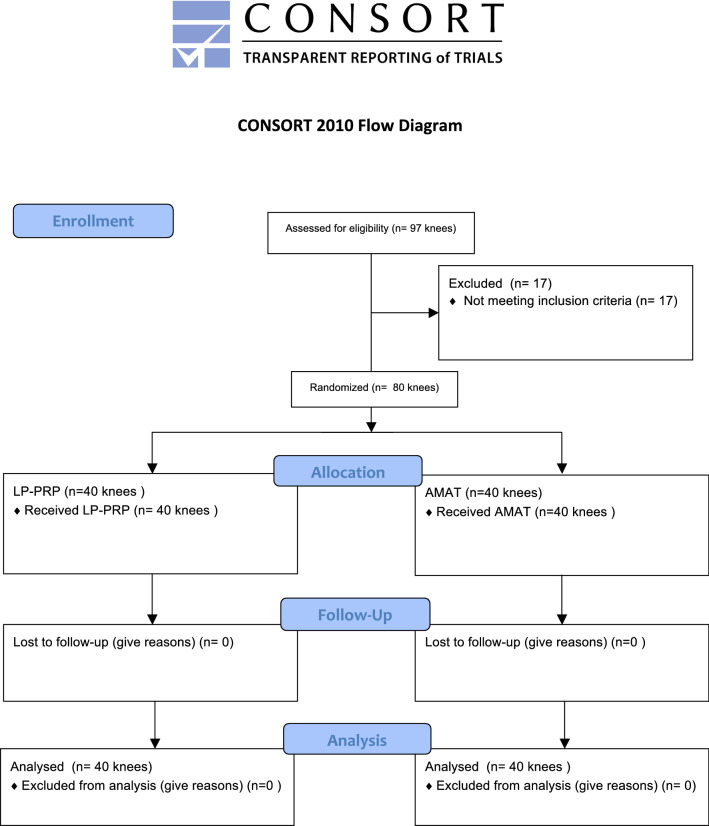
Flowchart showing the patients assessed for eligibility, excluded, enrolled, and analyzed in the study

Seventeen patients were excluded: four had severe knee OA, two had done previous cartilage transplantation, one had hepatitis, two were with infection, one had intra-articular corticosteroids in the three months post treatment, four were smokers, two had inflammatory arthritis, and one had severe cardiovascular disease. Fifty patients for a total of eighty knees completed the entire follow-up, of which 40 were placed in the LP-PRP + HA group and 40 in AMAT group (Fig. 1) [30]. No pre-operative differences were found between the two groups regarding age, gender, alignment, and knee OA (*p* > 0.05). Detailed results are reported in Table 3.

**Table 3:** Characteristics of the patient study population

Variables	LP-PRP + HA *N* = 40	AMAT *N* = 40	Overall *N* = 80	*p*-value
*Gender*				
Female	18 (45.0)	23 (57.5)	41 (51.3)	0.2634
Male	22 (55.0)	17 (42.5)	39 (48.7)	
*Kellgren-Lawrence*				
Grade 1	15 (37.5)	18 (45)	33 (41.3)	0.501
Grade 2	25 (62.5)	22 (55)	47 (58.7)	
Alignment	0.98° ± 2.36	1.08° ± 2.19	1.03° ± 2.26	0.844
*Age (preoperative)*				
< 65 years	23 (57.50)	24 (60.0)	47 (58.7)	0.8203
≥ 65 years	17 (42.50)	16 (40.0)	33 (41.3)	
Age (preoperative	62.00 ± 10.82	62.75 ± 12.99	62.38 ± 11.88	0.7797

Values are presented as *n* (%) or mean ± SD

LP-PRP + HA = leucocyte-poor platelet-rich plasma plus hyaluronic acid (HA); AMAT—autologous microfragmented adipose tissue

*Statistical significant difference

### Patient demographic

Between group comparability at baseline was found in age, gender, alignment degree, Kellgren-Lawrence OA degree, and all clinical scores except VAS and Marx Score. Baseline demographic and comparability is shown in Table 3. Clinical outcomes are reported in Table 4.

**Table 4:** Clinical Results for all patients, in LP-PRP + HA and AMAT group, respectively, at T_0_, T_1_, T_2_, and T_3_

Score	Groups		Comparison between groups (*p*-value)	Comparison between time points within the group (*p*-value)					
	LP-PRP + HA *N* = 40 Mean ± SD	AMAT *N* = 40 Mean ± SD							
				LP-PRP + HA			AMAT		
*TEGNER*									
T_0_	2.88 ± 1.47	3.48 ± 2.06	0.1551	T_0_	T_1_	T_2_	T_0_	T_1_	T_2_
T_1_	2.78 ± 1.48	3.33 ± 2.03	0.2841	1.0000	−	−	1.0000	−	−
T_2_	3.10 ± 1.79	3.28 ± 1.95	0.5729	1.0000	0.3428	−	1.0000	1.0000	−
T_3_	3.15 ± 1.56	3.50 ± 2.00	0.4889	0.5466	0.3476	1.0000	1.0000	1.0000	1.0000
*MARX*									
T_0_	1.78 ± 3.91	3.35 ± 4.91	0.0125*	T_0_	T_1_	T_2_	T_0_	T_1_	T_2_
T_1_	1.98 ± 3.74	1.78 ± 3.48	0.7916	1.0000	−	−	0.2499	−	−
T_2_	2.85 ± 4.54	2.35 ± 3.98	0.6922	1.0000	0.4688	−	1.0000	0.3750	−
T_3_	3.75 ± 5.16	2.33 ± 3.95	0.3408	0.0073*	0.0352*	1.0000	1.0000	0.8320	1.0000
*IKDC*									
T_0_	55.34 ± 15.21	48.26 ± 17.27	0.0554	T_0_	T_1_	T_2_	T_0_	T_1_	T_2_
T_1_	56.73 ± 19.71	55.87 ± 18.62	0.8416	1.0000	−	−	0.0103*	−	−
T_2_	62.68 ± 17.79	57.62 ± 19.22	0.2256	0.2044	0.1834	−	0.0169*	1.0000	−
T_3_	57.42 ± 21.96	55.60 ± 19.48	0.6961	1.0000	1.0000	0.3340	0.2152*	1.0000	1.0000
*KOOS symptoms*									
T_0_	73.45 ± 12.48	66.55 ± 17.63	0.1214	T_0_	T_1_	T_2_	T_0_	T_1_	T_2_
T_1_	74.63 ± 15.63	75.80 ± 17.98	0.5447	1.0000	−	−	0.0011*	−	−
T_2_	77.30 ± 13.41	75.30 ± 17.36	0.6447	1.0000	1.0000	−	0.0106*	1.0000	−
T_3_	75.33 ± 16.13	72.45 ± 17.55	0.5392	1.0000	1.0000	1.0000	0.2338	1.0000	1.0000
*KOOS pain*									
T_0_	74.60 ± 16.66	67.20 ± 17.92	0.0418*	T_0_	T_1_	T_2_	T_0_	T_1_	T_2_
T_1_	77.40 ± 18.63	76.22 ± 20.16	0.9425	1.0000	−	−	0.0217*	−	−
T_2_	73.78 ± 17.50	76.85 ± 21.19	0.3328	1.0000	1.0000	−	0.0412*	0.6026	−
T_3_	76.83 ± 20.96	72.83 ± 22.01	0.4838	1.0000	1.0000	1.0000	1.0000	1.0000	0.4009
*KOOS ADL*									
T_0_	77.15 ± 17.78	68.10 ± 20.22	0.0539	T_0_	T_1_	T_2_	T_0_	T_1_	T_2_
T_1_	76.45 ± 18.83	78.98 ± 18.78	0.5143	1.0000	−	−	0.0025*	−	−
T_2_	78.15 ± 17.19	81.45 ± 19.05	0.1987	1.0000	1.0000	−	< .0001*	0.6674	−
T_3_	65.68 ± 23.62	79.60 ± 20.20	0.0055*	0.0378*	0.0875	0.0069*	0.0150*	1.0000	1.0000
*KOOS sports*									
T_0_	44.05 ± 20.87	36.55 ± 28.20	0.0795	T_0_	T_1_	T_2_	T_0_	T_1_	T_2_
T_1_	40.95 ± 26.01	46.65 ± 31.23	0.4396	1.0000	−	−	0.2715	−	−
T_2_	43.88 ± 29.30	48.15 ± 30.00	0.7258	1.0000	1.0000	−	0.0722	1.0000	−
T_3_	68.35 ± 30.39	50.30 ± 30.15	0.0068*	0.0002*	< .0001*	< .0001*	0.0147*	0.6904	1.0000
*KOOS QOL*									
T_0_	46.78 ± 20.86	39.08 ± 25.02	0.1218	T_0_	T_1_	T_2_	T_0_	T_1_	T_2_
T_1_	55.98 ± 19.72	52.98 ± 25.96	0.5011	0.0326*	−	−	0.0180*	−	−
T_2_	57.00 ± 23.25	54.63 ± 24.55	0.6409	0.0347*	1.0000	−	0.0005*	1.0000	−
T_3_	56.28 ± 23.56	54.23 ± 26.00	0.6870	0.1263	1.0000	1.0000	0.0035*	1.0000	1.0000
*VAS*									
T_0_	3.85 ± 1.68	5.03 ± 2.02	0.0056*	T_0_	T_1_	T_2_	T_0_	T_1_	T_2_
T_1_	3.21 ± 2.08	3.77 ± 2.24	0.2179	0.3940	−	−	0.0005*	−	−
T_2_	2.64 ± 2.00	3.89 ± 2.51	0.0161*	0.0200*	0.0201*	−	0.0240*	1.0000	−
T_3_	3.35 ± 2.03	3.95 ± 2.59	0.3633	0.2871	1.0000	0.4489	0.0320*	1.0000	1.0000

LP-PRP + HA = leucocyte-poor platelet-rich plasma plus hyaluronic acid (HA); AMAT—autologous microfragmented adipose tissue; VAS—Visual Analogue Scale for pain; IKDC—International Knee Documentation Committee; KOOS—Knee Injury and Osteoarthritis Outcome Score; ADL—Activities of daily living; QOL—Quality of Life

^*^Statistical significant difference

### Results synthesis

The AMAT groups showed significantly higher pre-operative Marx score (3.35 ± 4.91 vs. 1.78 ± 3.91) and VAS score (5.03 ± 2.02 vs. 3.85 ± 1.68) (*p* < 0.05), higher VAS (3.89 ± 2.51 vs. 2.64 ± 2.00) at T_2_ and KOOS-ADL (79.60 ± 20.20 vs. 65.68 ± 23.62), and lower KOOS-Sports (50.30 ± 30.15 vs. 68.35 ± 30.39) at T_3_ (*p* < 0.05). Detailed results are reported in Table 4.

## Subgroup analysis

### Gender

#### Female

Female patients in the AMAT group reported a higher pre-operative Marx score, a higher VAS at T_2_, and a higher KOOS-ADL at the final follow-up (*p* < 0.05). In the LP-PRP + HA group, a significant improvement was noted for IKDC, KOOS-ADL, KOOS-Sports, KOOS-QOL, and VAS (*p* < 0.05). A significant improvement was noted in the AMAT group for Marx score, KOOS-symptoms, KOOS-ADL, KOOS-Sports, KOOS-QOL, and VAS (*p* < 0.05). Detailed results are reported in Appendix.

#### Male

Male patients in the AMAT group reported a lower pre-operative KOOS-Pain, KOOS-ADL, KOOS-Sports, KOOS-QOL, higher VAS, and a lower Marx score at the final follow-up (*p* < 0.05). In the LP-PRP + HA group, an improvement was noted for Marx score and KOOS-Sports, while in the AMAT group, only VAS at T_1_ showed a significant improvement (*p* < 0.05). Detailed results are reported in Appendix.

#### Age

##### Patients < 65 years of age

Young patients in the AMAT group showed a higher pre-operative Marx score, a lower pre-operative KOOS-ADL, and at final follow-up, a higher KOOS-ADL and lower KOOS-Sports (*p* < 0.05). Young patients in the LP-PRP + HA group reported significant improvement regarding Marx score, KOOS-ADL, and KOOS-Sports. In contrast, patients in the AMAT group showed significant improvement in IKDC, KOOS-symptoms, KOOS-pain, KOOS-ADL, KOOS-Sports, and VAS (*p* < 0.05). Detailed results are reported in Appendix.

##### Patients ≥ 65 years of age

The only difference between the two groups involved VAS at each time-point, with a significantly higher value in the AMAT group (*p* < 0.05). LP-PRP + HA patients reported significant improvement in KOOS-Pain and KOOS-Sports (*p* < 0.05), while no improvement was noted in the AMAT group (*p* > 0.05). Detailed results are reported in Appendix.

### Adverse reactions

No patient from either group had experienced major adverse effects from the injection or during the final follow-up. In the LP-PRP group 12 (30%) patients presented swelling, redness, and mild pain for one day after injection and two patients had synovitis for two days and required paracetamol and local ice. In AMAT group 5 (12.5%) patients had ecchymosis and bruising at the fat aspiration site for three days. No statistical difference in rate of complications was found between the two groups (*p* = 0.06).

## Discussion

The main findings of the current study found that both treatments lead to significant clinical improvement in several parameters with only slight differences were between the two different treatments. These results have also been confirmed by performing a subgroup analysis, by age and gender, confirming the treatments’ efficacy without significant difference among the two groups regardless of age and gender. The two groups were homogeneous as regarding all pre-operative scores, but it is interesting to note that early-mid-term follow-up (T_2_) LP-PRP reported a lower VAS score, confirming how the three injections can lead to a beneficial long-lasting effect [13].

This study has some limitations; it was not possible to conduct a prospective randomized and double-blinded study for ethical and practical reasons, as PRP does not require liposuction.

Furthermore, to avoid bias in the study, several restricted inclusion and exclusion criteria were followed, starting from age; in fact, the number of chondrocytes and bone-marrow-derived MSCs and their proliferative and matrix-forming potential may decrease as the years go by [31]. This might reduce the healing capacity of cartilage in older patients, and this is a common exclusion criterion [31]. In addition, patients with signs of joint-wide OA were excluded.

Finally, we excluded patients with BMI greater than 30, tricompartmental OA, inflammatory arthritis, previous cartilage transplantation, and ligamentous instability [31]. All these comorbidities or previous surgeries can increase the risk of excessive focal or abnormal loading and the likelihood of degeneration. Active inflammatory processes would be expected to interfere with any repair status and could limit the efficacy of tissue engineering strategies [31].

Another limitation is the lack of imaging evaluation that we tried to overcome with exhaustive clinical scores repeated over time. The study presents mid-term clinical results and a long follow-up may be necessary to confirm these results. The study did not include a placebo control group to compare results, as it is not ethically acceptable in the institution. More extensive research with long-term follow-up and biological outcomes will be of great interest for future studies.

The development of these new regenerative procedures opens up interesting scenarios in treating mild cases of arthrosis, where surgery has no place but has a significant social and QOL impact [32n]. OA is one of the leading causes of functional impairment in daily living activities among older adults and a serious issue in public health throughout the world. Pain and limitations result primarily from mechanical changes in the knee [32]. Patients with OA may also suffer from various psychological problems such as sleep disturbance, depressive mood, and individual’s subjective assessment of their mental and physical well-being manifested by health-related QOL [33, 34].

Currently, only a few studies have analyzed the efficacy of PRP in association with HA, reporting good to excellent outcomes; Saturveithan et al. in 2015 analyzed the efficacy of PRP in association with HA in knee OA of grades III and IV, reporting improvement in terms of functional outcome and pain for up to six months [35].

In 2017, Yu et al. treated more than 350 patients with knee OA randomizing into four blinded different groups: PRP (2–14 ml), HA (0.1–0.3 mg), PRP plus HA, and placebo groups [36]. At the final follow-up, the authors confirmed that PRP in association with HA significantly improved pain, reduced cellular immune responses, and promoted angiogenesis, with beneficial effects on histological parameters compared with PRP or HA treatment alone [36].

Recently, Lana et al. confirmed previous findings, analyzing 105 patients with moderate knee OA and randomized to one of three interventions: HA (*n* = 36), PRP (*n* = 36), or HA + PRP (*N* = 33) [37]. The combination of HA plus PRP resulted in better outcomes than isolated HA for up to one year and isolated PRP for up to three months.

Despite the demonstrated efficacy of PRP, there remains a great deal of doubt about its classification and preparation, making it difficult to analyze clinical outcomes.

Multiple variables comprise the formulations of PRP, with the predominant categories involving platelet concentration, white blood cell concentration, and growth factor quantity [38–40]. Riboh et al. performed a meta-analysis of the current literature and found LP-PRP resulted in significantly better functional outcomes when compared with LR-PRP [41].

In the other groups, treatment of patients with AMAT was decided; in fact, these cells contain immunomodulatory properties, making them a promising candidate for OA’s regenerative treatment [7]. The ADSCs in the SVF secrete several anti-inflammatory substances such as IL-1RA, nitric oxide, TGF β1, SDF-1, and LL37, among others. These alleviate the inflammatory state and relieve in the affected joint [7].

Furthermore, AMAT is present in a huge amount in the human body (more than 5% of nucleated cells in adipose tissue), with the relative simplicity of harvesting and lower donor-site morbidity, and its rapid expansion and high proliferative capabilities [7]. Moreover, ADSCs are able to maintain their features even if manipulated through different cell cultures compared to different cell lines [7].

A recent systematic review reported good to excellent clinical results after AMAT injection, with minimal complication rates [42]. Gobbi et al., in a multi-centric, international, and open-label study published in 2021, show that a single-dose of AMAT injection leads to clinical and functional, improvement at two years in seventy-five patients with KL grades two, three or four [43].

## Conclusions

AMAT did not show significant superior clinical improvement compared with three LP-PRP combined with HA injections in terms of functional improvement at different follow-up points. Both procedures were safe with no major complications reporting good results at mid-term follow-up, improving knee function, pain, and quality of live regardless of age and gender.

## Supplementary Information

Below is the link to the electronic supplementary material.

Supplementary file1 (XLSX 33 kb)

## Data Availability

Raw data have been submitted as supplementary material to the journal.

## Declarations

### Study registration

Researchregistry6530—www.researchregistry.com.

## References

[1] Naik A, Shanmugasundaram S, Mahadev K, Shetty AA, Kim SJ (2021) Volume index as a new measure of cartilage loss: a retrospective MRI-based study of chondral injury patterns in adult patients with knee pain. Eur J Orthop Surg Traumatol 2021. 10.1007/s00590-021-03158-y 10.1007/s00590-021-03158-y 34743222

[2] Hansen L, Larsen P, Elsoe R (2022). Characteristics of patients requiring early total knee replacement after surgically treated lateral tibial plateau fractures—a comparative cohort study. Eur J Orthop Surg Traumatol.

[3] Clementi D, D'Ambrosi R, Bertocco P, Bucci MS, Cardile C, Ragni P, Giaffreda G, Ragone V (2018). Efficacy of a single intra-articular injection of ultra-high molecular weight hyaluronic acid for hip osteoarthritis: a randomized controlled study. Eur J Orthop Surg Traumatol.

[4] Hohmann E, Tetsworth K, Glatt V (2020). Is platelet-rich plasma effective for the treatment of knee osteoarthritis? A systematic review and meta-analysis of level 1 and 2 randomized controlled trials. Eur J Orthop Surg Traumatol.

[5] Madry H, Kon E, Condello V, Peretti GM, Steinwachs M, Seil R, Berruto M, Engebretsen L, Filardo G, Angele P (2016). Early osteoarthritis of the knee. Knee Surg Sports Traumatol Arthrosc.

[6] Lee WS, Kim HJ, Kim KI, Kim GB, Jin W (2019). Intra-articular injection of autologous adipose tissue-derived mesenchymal stem cells for the treatment of knee osteoarthritis: a phase IIb, randomized, placebo-controlled clinical trial. Stem Cells Transl Med.

[7] Usuelli FG, D'Ambrosi R, Maccario C, Indino C, Manzi L, Maffulli N (2017). Adipose-derived stem cells in orthopaedic pathologies. Br Med Bull.

[8] Dallo I, Szwedowski D, Mobasheri A, Irlandini E, Gobbi A (2021). A Prospective study comparing leukocyte-poor platelet-rich plasma combined with hyaluronic acid and autologous microfragmented adipose tissue in patients with early knee osteoarthritis. Stem Cells Dev.

[9] Schulz KF, Altman DG, Moher D; CONSORT Group (2010). CONSORT 2010 statement: updated guidelines for reporting parallel group randomised trials. BMJ.

[10] Mast J, Vanermen F, Van de Vyver A, Nicolai P (2022) The effect of gender, age, BMI and Kellgren-Lawrence grade on functional outcome after Physica ZUK medial unicompartmental knee replacement. Eur J Orthop Surg Traumatol. Doi: 10.1007/s00590-022-03202-5. 10.1007/s00590-022-03202-5 35119488

[11] Jones C, Nawaz Z, Hassan A, White S, Khaleel A (2016). The variability in the external rotation axis of the distal femur: an MRI-based anatomical study. Eur J Orthop Surg Traumatol.

[12] Rosenberger WF, Uschner D, Wang Y (2019). Randomization: The forgotten component of the randomized clinical trial. Stat Med.

[13] Gobbi A, Lad D, Karnatzikos G (2015). The effects of repeated intra-articular PRP injections on clinical outcomes of early osteoarthritis of the knee. Knee Surg Sports Traumatol Arthrosc.

[14] Dohan Ehrenfest DM, Rasmusson L, Albrektsson T (2009). Classification of platelet concentrates: from pure platelet-rich plasma (P-PRP) to leucocyte- and platelet-rich fibrin (L-PRF). Trends Biotechnol.

[15] DeLong JM, Russell RP, Mazzocca AD (2012). Platelet-rich plasma: the PAW classification system. Arthroscopy.

[16] Abate M, Verna S, Schiavone C, Di Gregorio P, Salini V (2015). Efficacy and safety profile of a compound composed of platelet-rich plasma and hyaluronic acid in the treatment for knee osteoarthritis (preliminary results). Eur J Orthop Surg Traumatol.

[17] Abbassy AA, Trebinjac S, Kotb N (2020). The use of cellular matrix in symptomatic knee osteoarthritis. Bosn J Basic Med Sci.

[18] Mazzucco L, Balbo V, Cattana E, Guaschino R, Borzini P (2009). Not every PRP-gel is born equal. Evaluation of growth factor availability for tissues through four PRP-gel preparations: fibrinet, RegenPRP-Kit, Plateltex and one manual procedure. Vox Sang.

[19] Caplan AI (2017) Mesenchymal stem cells: time to change the name! Stem Cells Transl Med 6:1445–1451 10.1002/sctm.17-0051 PMC5689741 28452204

[20] Panchal J, Malanga G, Sheinkop M (2018) Safety and efficacy of percutaneous injection of lipogems micro-fractured adipose tissue for osteoarthritic knees. Am J Orthop (Belle Mead NJ) 47(11) 10.12788/ajo.2018.0098 30517209

[21] Russo A, Condello V, Madonna V, Guerriero M, Zorzi C (2017). Autologous and micro-fragmented adipose tissue for the treatment of diffuse degenerative knee osteoarthritis. J Exp Orthop.

[22] Gay C, Chabaud A, Guilley E, Coudeyre E (2016). Educating patients about the benefits of physical activity and exercise for their hip and knee osteoarthritis. Systematic literature review. Ann Phys Rehabil Med.

[23] Collins NJ, Misra D, Felson DT, Crossley KM, Roos EM (2011). Measures of knee function: international knee documentation committee (IKDC) subjective knee evaluation form, knee injury and osteoarthritis outcome score (KOOS), knee injury and osteoarthritis outcome score physical function short form (KOOS-PS), knee outcome survey activities of daily living scale (KOS-ADL), Lysholm knee scoring scale, oxford knee score (OKS), Western Ontario and McMaster Universities Osteoarthritis Index (WOMAC), activity rating scale (ARS), and tegner activity score (TAS). Arthritis Care Res (Hoboken).

[24] Karcioglu O, Topacoglu H, Dikme O, Dikme O (2018). A systematic review of the pain scales in adults: Which to use?. Am J Emerg Med.

[25] Shirazi CP, Israel HA, Kaar SG (2016). Is the Marx activity scale reliable in patients younger than 18 years?. Sports Health.

[26] Tegner Y, Lysholm J (1985). Rating systems in the evaluation of knee ligament injuries. Clin Orthop Relat Res.

[27] Van Genechten W, Vuylsteke K, Martinez PR, Swinnen L, Sas K, Verdonk P (2021). Autologous micro-fragmented adipose tissue (MFAT) to treat symptomatic knee osteoarthritis: early outcomes of a consecutive case series. J Clin Med.

[28] Willhuber GC, Stagnaro J, Petracchi M, Donndorff A, Monzon DG, Bonorino JA, Zamboni DT, Bilbao F, Albergo J, Piuzzi NS, Bongiovanni S (2018). Short-term complication rate following orthopedic surgery in a tertiary care center in Argentina. SICOT J.

[29] Vickerstaff V, Omar RZ, Ambler G (2019). Methods to adjust for multiple comparisons in the analysis and sample size calculation of randomised controlled trials with multiple primary outcomes. BMC Med Res Methodol.

[30] Andrade C (2015). Examination of participant flow in the CONSORT diagram can improve the understanding of the generalizability of study results. J Clin Psychiatry.

[31] Martín AR, Patel JM, Zlotnick HM, Carey JL, Mauck RL (2019). Emerging therapies for cartilage regeneration in currently excluded 'red knee' populations. NPJ Regen Med.

[32] Bouras T, Tzanos IA, Forster M, Panagiotopoulos E (2021). Correlation of quality of life with instrumented analysis of a total knee arthroplasty series at the long-term follow-up. Eur J Orthop Surg Traumatol.

[33] Ahn H, Weaver M, Lyon D, Choi E, Fillingim RB (2017). Depression and pain in Asian and White Americans with knee osteoarthritis. J Pain.

[34] Mahdi A, Hälleberg-Nyman M, Wretenberg P (2021). Reduction in anxiety and depression symptoms one year after knee replacement: a register-based cohort study of 403 patients. Eur J Orthop Surg Traumatol.

[35] Saturveithan C, Premganesh G, Fakhrizzaki S, Mahathir M, Karuna K, Rauf K, William H, Akmal H, Sivapathasundaram N, Jaspreet K (2016). Intra-articular hyaluronic acid (HA) and platelet rich plasma (PRP) injection versus hyaluronic acid (HA) injection alone in patients with grade iii and iv knee osteoarthritis (OA): a retrospective study on functional outcome. Malays Orthop J.

[36] Yu W, Xu P, Huang G, Liu L (2018). Clinical therapy of hyaluronic acid combined with platelet-rich plasma for the treatment of knee osteoarthritis. Exp Ther Med.

[37] Lana JF, Weglein A, Sampson SE, Vicente EF, Huber SC, Souza CV, Ambach MA, Vincent H, Urban-Paffaro A, Onodera CM, Annichino-Bizzacchi JM, Santana MH, Belangero WD (2016). Randomized controlled trial comparing hyaluronic acid, platelet-rich plasma and the combination of both in the treatment of mild and moderate osteoarthritis of the knee. J Stem Cells Regen Med.

[38] Mochizuki T, Yano K, Ikari K, Hiroshima R, Kawakami K, Koenuma N, Ishibashi M, Shirahata T, Momohara S (2016). Platelet-rich plasma for the reduction of blood loss after total knee arthroplasty: a clinical trial. Eur J Orthop Surg Traumatol.

[39] Jang SJ, Kim JD, Cha SS (2013). Platelet-rich plasma (PRP) injections as an effective treatment for early osteoarthritis. Eur J Orthop Surg Traumatol.

[40] Lee GW, Son JH, Kim JD, Jung GH (2013). Is platelet-rich plasma able to enhance the results of arthroscopic microfracture in early osteoarthritis and cartilage lesion over 40 years of age?. Eur J Orthop Surg Traumatol.

[41] Riboh JC, Saltzman BM, Yanke AB, Fortier L, Cole BJ (2016). Effect of leukocyte concentration on the efficacy of platelet-rich plasma in the treatment of knee osteoarthritis. Am J Sports Med.

[42] Biazzo A, D'Ambrosi R, Masia F, Izzo V (2020). Verde F (2020) Autologous adipose stem cell therapy for knee osteoarthritis: where are we now?. Phys Sportsmed.

[43] Gobbi A, Dallo I, Rogers C, Striano RD, Mautner K, Bowers R, Rozak M, Bilbool N, Murrell WD (2021). Two-year clinical outcomes of autologous microfragmented adipose tissue in elderly patients with knee osteoarthritis: a multi-centric, international study. Int Orthop.

